# A derivative-free root-finding algorithm using exponential method and its implementation

**DOI:** 10.1186/s13104-023-06554-1

**Published:** 2023-10-17

**Authors:** Srinivasarao Thota, Mohamed M. Awad, P. Shanmugasundaram, Laxmi Rathour

**Affiliations:** 1 Department of Mathematics, Amrita School of Physical Sciences, Amrita Vishwa Vidyapeetham, Amaravati, Andhra Pradesh 522503 India; 2 Mechanical Power Engineering Department, Faculty of Engineering, Mansoura University, Mansoura, 35516 Egypt; 3 Department of Mathematics, College of Natural & Computational Sciences, Mizan-Tepi University, Mizan Teferi, Ethiopia; 4 Department of Mathematics, National Institute of Technology, Chaltlang, 796012 Aizawl, Mizoram India

**Keywords:** Iterative algorithms, Nonlinear equations, Exponential method, Algorithm implementation, Root-finding algorithms, 65H04, 65Hxx

## Abstract

**Objective:**

In this paper, we develop a new root-finding algorithm to solve the given non-linear equations. The proposed root-finding algorithm is based on the exponential method. This algorithm is derivative-free and converges fast.

**Results:**

Several numerical examples are presented to illustrate and validation of the proposed methods. Microsoft Excel and Maple implementation of the proposed algorithm is presented with sample computations.

## Introduction

Finding an approximate root of non-linear equations using iterative algorithms plays a significant role in the computational and applied mathematics. The applications of non-linear equations of the type $f(x) = 0$ arise in various branches of scientific computing fields. Solving such non-linear equations is one of the most important problems and frequently appearing in different scientific fields that can be modeled through nonlinear equations. In recent time, several researchers, engineers and scientists focused on solving non-linear equations numerically as well as analytically. Iterative algorithms play a vital role in finding the solution of such non-linear problems. In general, the roots of non-linear or transcendental equations cannot be expressed in closed form or cannot be computed analytically. The root-finding algorithms provide us to compute approximations to the roots; these approximations are expressed either as small isolating intervals or as floating point numbers. There are various numerical algorithm/methods available in the literature, see for example [1–20], for more details.

Many new modified/hybrid/multi-step iterative algorithms are developed in the last few years, by employing various mathematical algorithms/techniques. Noor et al. discussed the fifth-order second derivative-free algorithm in 2007, see [21], by using the finite difference scheme. Grau-Sanchez et al. presented a fifth-order Chebyshev-Halley type method in 2008, see [22]. Zhanlav et al. proposed a three- step fifth-order iterative algorithm in 2010 [23]. Nazeer et al. introduced a novel second derivative-free Householder’s method having fifth-order convergence by using finite-difference scheme [24] in 2016. Recently, in 2021, Amir et al. developed an efficient and derivative-free algorithm for determining an approximate solution of the given non-linear scalar equations by applying forward- and finite-difference schemes similar to Traub’s method, see [25]. In this paper, we propose a new root- finding algorithm, which is derivative-free, using exponential method. To propose the algorithm with derivative-free, we employ the forward difference scheme and finite difference scheme. This gives computationally low cost. Microsoft Excel and Maple implementations of the proposed algorithm are presented. Maple and Excel implementations with sample computations for differential and transcendental equations are available in the literature, see for example [18, 19, 26, 27] and there are various techniques for different type of applications, see [20, 28–35], the references cited therein.

### Preliminaries

In this paper, we consider the non-linear equation of the type

1 $$ f(x) = 0. $$

Iterations techniques are a common approach widely used in various numerical algorithms/methods. It is a hope that an iteration in the general form of *x*_*n*+1_ = *g*(*x*_*n*_) will eventually converge to the true solution *α* of the problem (1) at the limit when *n* → ∞. The concern is whether this iteration will converge, and, if so, the rate of convergence. Specifically we use the following expression to represent how quickly the error *e*_*n*_ = *α* − *x*_*n*_ converges to zero. Let *e*_*n*_ = *α* − *x*_*n*_ and *e*_*n*+1_ = *α* − *x*_*n*+1_ for *n* ≥ 0 be the errors at *n*-th and (*n* + 1)-th iterations respectively. If two positive constants *µ* and *p* exist, and

2 $$ \mathop {\lim }\limits_{n \to \infty } \frac{{|e_{n + 1} |}}{{|e_{n} |^{p} }} = \frac{{|\alpha - x_{n + 1} |}}{{|\alpha - x_{n} |^{p} }} = \mu , $$

then the sequence is said to converge to *α*. Here *p* ≥ 1 is called the *order of convergence*; the constant *µ* is the *rate of convergence* or *asymptotic error constant*. This expression may be better understood when it is interpreted as |*e*_*n*+1_|= *µ*|*e*_*n*_|^*p*^ when *n* → ∞. Obviously, the larger *p* and the smaller *µ*, the more quickly the sequence converges.

#### **Theorem 1**

 [16, 36] *Suppose that* $g \in C^{p} [a,b]$*. If g*^(*k*)^(*x*) = 0*, for k* = 0*,* 1*,* 2*,..., p* − 1 *and* $g^{(p)} (x) \ne 0$*, then the sequence* {*x*_*n*_} *is of order p.*

This paper focuses on developing iterative algorithm having fourth-order of convergence. The following section presents the proposed algorithm using Newton–Raphson method and exponential method without computing the derivative.

## Main text (a new iterative algorithm)

We assume that *α* is an exact root of the Eq. (1) and let *a* be an initial approximation (sufficiently close) to *α*. In the exponential method, we can find first approximation root using the following formula. See [5] for more details.

$$ x = a \exp \left( {\frac{ - f(a)}{{af^{\prime}(a)}}} \right). $$

If *x*_*n*+1_ is the required root, then the exponential formula can be expressed as, for *n* = 0*,* 1*,* 2*,* 3*,...*,

3 $$ x_{n + 1} = x_{n} \exp \left( {\frac{{ - f(x_{n} )}}{{x_{n} f^{\prime}(x_{n} )}}} \right). $$

which has more than second-order convergence.

Suppose *y*_*n*_ = *x*_*n*+1_, where *x*_*n*+1_ is the Newton–Raphson formula, is predictor and corrector, then Traub [37] created a new two-step iterative algorithm as follows, *n* = 0*,* 1*,* 2*,* 3*,...*,

$$ x_{n + 1} = y_{n} - \frac{{f(y_{n} )}}{{f^{\prime}(y_{n} )}}. $$

It is shown in [37] that the Traub’s method has fourth-order convergence. Since Newton–Raphson formula repeated twice, the Traub’s method includes four computations to execute the algorithm. Amir et al. extended the Traub’s method to derivative-free algorithm by applying forward- and finite-difference schemes on Traub’s method.

In this paper, we propose a new two-step iterative algorithm similar to that of Amir et al., and the proposed algorithm has more than fourth-order convergence. The proposed method is created using the exponential method designed by Thota et al. [5]. Using exponential method, one can obtain an approximate root of a given non-linear equation using the formula (3). The order of convergence of the exponentiation method is more than two, see [5] for more details. Using exponential method (3), the proposed algorithm consists of the following steps:

4 $$ \begin{gathered} y_{n} = x_{n} \exp \left( {\frac{{ - f(x_{n} )}}{{x_{n} f^{\prime}(x_{n} )}}} \right), \hfill \\ x_{n + 1} = y_{n} \exp \left( {\frac{{ - f(y_{n} )}}{{y_{n} f^{\prime}(y_{n} )}}} \right). \hfill \\ \end{gathered} $$

One can observe that, this is a two-step iteration method to calculate roots of a given non-linear equations. Since there are two steps in the algorithm and it required four evaluations for its execution. The biggest disadvantage of the algorithm (4) is computational cost of each iteration which is more. In order to reduce the high computational cost, we replace the first derivative by approximation and this suggests a novel derivative-free algorithm. Hence, it can be applied easily to the given non- linear equations where the first derivative is not defined in the domain. We use the forward difference approximation in the predictor to approximate the first derivative as follows, here *f* (*x*_*n*_) ≥ 0,

5 $$ f^{\prime}(x_{n} ) = \frac{{f(x_{n} + f(x_{n} )) - f(x_{n} )}}{{f(x_{n} )}} = g(x_{n} ). $$

Now, we use finite difference approximation in the corrector step (i.e., in step 2) as follows

6 $$ f^{\prime}(y_{n} ) = \frac{{f(y_{n} ) - f(x_{n} )}}{{y_{n} - x_{n} }} = h(x_{n} ,y_{n} ). $$

Substituting the Eqs. (5)–(6) in algorithm (4), we obtain a new efficient and derivative-free iterative algorithm to calculate the approximate solution of a given non-linear equation as follows

7 $$ \begin{gathered} y_{n} = x_{n} \exp \left( {\frac{{ - f(x_{n} )}}{{x_{n} g(x_{n} )}}} \right), \hfill \\ x_{n + 1} = y_{n} \exp \left( {\frac{{ - f(y_{n} )}}{{y_{n} h(x_{n} ,y_{n} )}}} \right), \hfill \\ \end{gathered} $$

where *g*(*x*_*n*_) and *h*(*x*_*n*_*, y*_*n*_) are as given (5)–(6). This is a new iterative algorithm to find a root of transcendental equations in two-step without involvement of any derivative. One of the advantages of the proposed algorithm is existence of root where the first derivative does not exist at some particular points in the domain, and another big advance is the computational complexity. This method has more than fourth order convergence and its convergence analysis is presented in the following section.

### Analysis of convergence

In this section, we show in the following theorem that the order of converges of the proposed algorithm is five. Let *I* ⊂ R be an open interval. To prove this, we follow the proofs of ([2], Theorem 5, Theorem 6) or ([16], Theorem 2, Theorem 3, Theorem 4).

#### **Theorem 2**

*Let f*: *I* → R*. Suppose α* ∈ *I is a simple root of* (1) *and θ is a sufficiently small neighborhood of α. Then the iterative formula* (7) *produces a sequence of iterations* {*x*_*n*_: *n* = 1*,* 2*,...*} *with order of convergence four.*

#### ***Proof***

Let.

$$ y = x \exp \left( {\frac{ - f(x)}{{xg}}} \right), {\text{and}} R(x) = y \exp \left( {\frac{ - f(y)}{{yh}}} \right), $$

where

$$ g = \frac{f(x + f(x)) - f(x)}{{f(x)}}, h = \frac{f(y) - f(x)}{{y - x}}. $$

Since *α* is a root of *f* (*x*), hence *f* (*α*) = 0. One can compute that

$$ \begin{gathered} R(\alpha ) = \alpha , \hfill \\ R^{\prime}(\alpha ) = 0, \hfill \\ R^{\prime\prime}(\alpha ) = 0, \hfill \\ R^{\prime\prime\prime}(\alpha ) = 0, \hfill \\ R^{iv} (\alpha ) \ne 0. \hfill \\ \end{gathered} $$

Hence the Algorithm (7) has fourth-order convergence, by Theorem 1. $\square$

One can also verify that the order of convergence of the proposed algorithm as in the following example.

#### ***Example 1***

Consider the following equation.

8 $$ f\left( x \right) = x^{{2}} - {1}{\text{.}} $$

It has a root* α* =  − 1. We show, as discussed in proof of Theorem 2, that the proposed algorithm has fourth-order convergence. Following Theorem 2, we have

$$\begin{gathered} g = \frac{f(x + f(x)) - f(x)}{{f(x)}} = x^{2} - 2x - 1, \hfill \\ y = x \exp \left( {\frac{ - f(x)}{{xg}}} \right) = xe^{{\frac{(x - 1)(x + 1)}{{x(x^{2} - 2x - 1)}}}} , \hfill \\ h = \frac{f(y) - f(x)}{{y - x}} = x\left( {e^{{ - \frac{(x - 1)(x + 1)}{{x(x^{2} - 2x - 1)}}}} + 1} \right), \hfill \\ R(x) = y \exp \left( {\frac{ - f(y)}{{yh}}} \right)\,\,\, = \,\, xe^{{\frac{{ - x^{4} e^{t} - 3x^{3} e^{t} + x^{2} e^{ - t} + x^{2} e^{t} - x^{3} + 2xe^{ - t} + xe^{t} - e^{ - t} + x}}{{x^{2} (x^{2} - 2x - 1)(e^{t} + 1)}}}} ,\, \hfill \\ \end{gathered}$$

where

$$ t = - \frac{(x - 1)(x + 1)}{{x(x^{2} - 2x - 1)}}. $$

Now

$$ \begin{gathered} R(\alpha ) = - 1 = \alpha , \hfill \\ R^{\prime}(\alpha ) = 0, \hfill \\ R^{\prime\prime}(\alpha ) = 0, \hfill \\ R^{\prime\prime\prime}(\alpha ) = 0, \hfill \\ R^{(iv)} (\alpha ) = 8 \ne 0. \hfill \\ \end{gathered} $$

Hence, by Theorem 2, the algorithm in (7) has fourth-order convergence.

### Numerical examples

#### ***Example 2***

Consider a transcendental equation $e^{x} + \cos (x) - 1 = 0$ with ${\text{x}}\_0 = - 2$. Now we can compute a real of the given equation using the proposed algorithm (7) as follows.

 Suppose $f(x) = e^{x} + \cos (x) - 1,$ then we have $g(x_{0} ) = \frac{{f(x_{0} + f(x_{0} )) - f(x_{0} )}}{{f(x_{0} )}} = 0.5246013002,$ $y_{0} = x_{0} \exp \left( {\frac{{ - f(x_{0} )}}{{x_{0} g(x_{0} )}}} \right) = - 0.5900190724,$ $h(x_{0} ,y_{0} ) = \frac{{f(y_{0} ) - f(x_{0} )}}{{y_{0} - x_{0} }} = 1.181617637$ and, $x_{1} = y_{0} \exp \left( {\frac{{ - f(y_{0} )}}{{y_{0} h(x_{0} ,y_{0} )}}} \right) = - 1.025295284.$

 Similarly, we have the values in iteration 2:

$$ \begin{gathered} g(x_{1} ) = \frac{{f(x_{1} + f(x_{1} )) - f(x_{1} )}}{{f(x_{1} )}} = 1.222059474, \hfill \\ y_{1} = x_{1} \exp \left( {\frac{{ - f(x_{1} )}}{{x_{1} g(x_{1} )}}} \right) = - 0.9298264088, \hfill \\ h(x_{1} ,y_{1} ) = \frac{{f(y_{1} ) - f(x_{1} )}}{{y_{1} - x_{1} }} = 1.205191949, \hfill \\ x_{2} = y_{1} \exp \left( {\frac{{ - f(y_{1} )}}{{y_{1} h(x_{1} ,y_{1} )}}} \right) = - 0.9237026911. \hfill \\ \end{gathered} $$

Iteration 3:

$$ \begin{gathered} g(x_{2} ) = \frac{{f(x_{2} + f(x_{2} )) - f(x_{2} )}}{{f(x_{2} )}} = 1.194895070, \hfill \\ y_{2} = x_{2} \exp \left( {\frac{{ - f(x_{2} )}}{{x_{2} g(x_{2} )}}} \right) = - 0.9236326626, \hfill \\ h(x_{2} ,y_{2} ) = \frac{{f(y_{2} ) - f(x_{2} )}}{{y_{2} - x_{2} }} = 1.194879228, \hfill \\ x_{3} = y_{2} \exp \left( {\frac{{ - f(y_{2} )}}{{y_{2} h(x_{2} ,y_{2} )}}} \right) = - 0.9236326590. \hfill \\ \end{gathered} $$

One can obtain the function value at *x*_3_ = − 0.9236326590 as *f* (− 0.9236326590) = − 5.3608 × 10^−11^. Hence the required root *x* = − 0.9236326590 is obtained in 3 iterations using the proposed algorithm.

#### ***Example 3***

Consider a polynomial equation to find a real root.

9 $$ 0.{986}x^{{3}} - {5}.{18}x^{{2}} + {9}.0{64}x - {5}.{287} = 0 $$

with *x*_0_ = 0*.*6. Following Example 2 using the proposed algorithm (7), we have Iteration 1:

$$ \begin{gathered} g\left( {x_{0} } \right) = 11.24874333, \hfill \\ y_{0} = 0.749437179, \hfill \\ h\left( {x_{0} ,y_{0} } \right) = 3.427685909, \hfill \\ x_{1} = 1.101280164383,\quad f\left( {x_{1} } \right) = - 0.270349537. \hfill \\ \end{gathered} $$

Other iterations values are

$$ \begin{gathered} x_{2} = 1.387799514358,\quad f\left( {x_{2} } \right) = - 0.048898877, \hfill \\ x_{3} = 1.568877491071,\quad f\left( {x_{3} } \right) = - 0.00884392, \hfill \\ x_{4} = 1.753077607303,\quad f\left( {x_{4} } \right) = - 0.004242231, \hfill \\ x_{5} = 1.883259728433,\quad f\left( {x_{5} } \right) = - 0.00298145, \hfill \\ x_{6} = 1.922476516171,\quad f\left( {x_{6} } \right) = - 0.000608725, \hfill \\ x_{7} = 1.929827783304,\quad f\left( {x_{7} } \right) = - 1.59531E - 06, \hfill \\ x_{8} = 1.929846242848,\quad f\left( {x_{8} } \right) = - 2.30926E - 14. \hfill \\ \end{gathered} $$

Hence, the required approximate root of the given equation (9) is *x* = 1.929846242848.

### Implementation of the proposed algorithm

####  Implementation in MS Excel

**Fig. 1:**
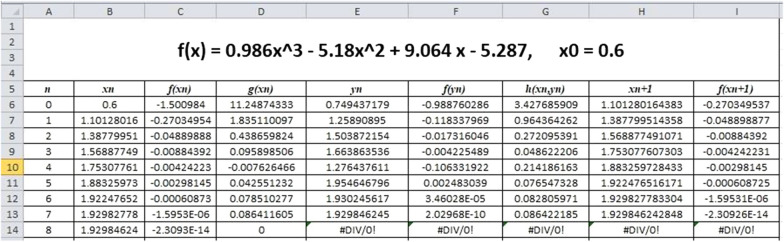
Proposed algorithm in Excel

The proposed method can be computed in Excel easily as follows. The number of iterations n, initial guess $x_{n} ,f\left( {x_{n} } \right),g\left( {x_{n} } \right),y_{n} ,f\left( {y_{n} } \right),h\left( {x_{n} ,{\text{ }}y_{n} } \right),x_{{n + 1}} \;{\text{and}}\;f\left( {x_{{n + 1}} } \right)$ are placed in Excel cells, for example, in A5, B5, C5, D5, E5, F5, G5, H5 and I5 respectively. Enter the respective values in 6th row, i.e., n = 0, x_0_, “= f(B6)”, “= (f(B6 + C6) − C6)/C6”, “= B6*EXP((− C6)/(B6 * D6))”, “= f(E6)”, “= (F6 − C6)/(E6 − B6)”, “= E6 * EXP((− F6)/(E6 * G6))” and “= f(H6)” respectively in A6–I6. For sec- ond iteration, we need to replace x_n_ by x_n+1_ in B7 using the command “= H6”. The last columns, C6–I6, are drag down to get next iteration value. Finally, drag down the entire 8th row, A7–I7, until the required number of iterations, see Fig. 1. Sample computations using MS Excel are presented in the following section.

##### ***Example 4***

Consider the Eq. (9) presented in Example 3 for sample computations using MS Excel.

$$ f\left( x \right) = 0.986x^{3}  - 5.181x^{2}  + 9.067x - 5.289 $$

with *x*_0_ = 0*.*6. Following the procedure in Section, we have the results as in Fig. 1*.*

#### Implementation in Maple


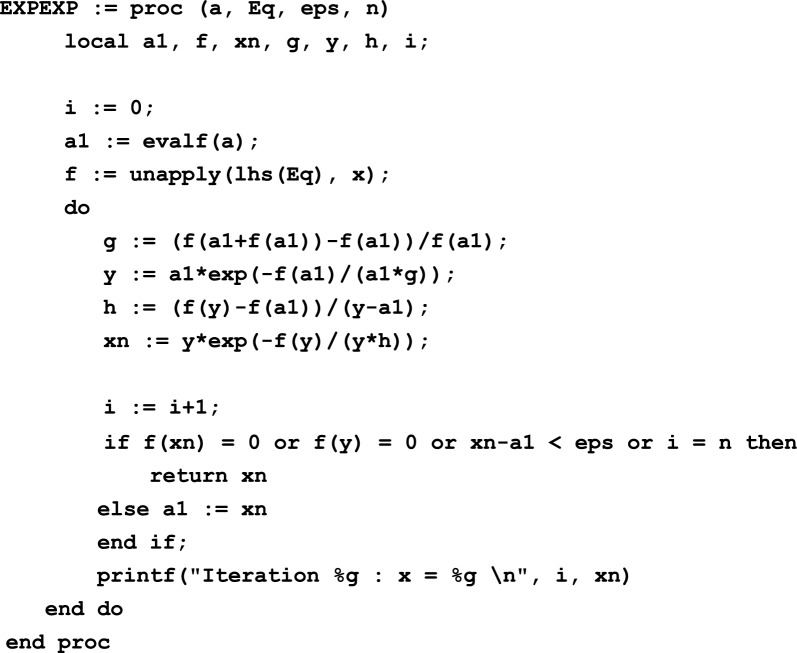


##### ***Example 5***

Consider the equation *e*^*x*^ + cos(*x*) − 1 = 0 given in Example 2 for sample computations in Maple.
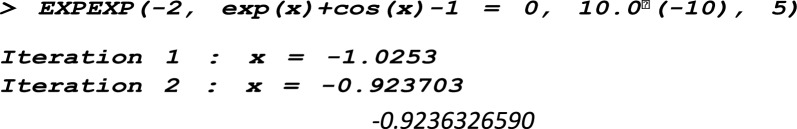


## Conclusion

In this paper, we proposed a new root-finding algorithm to solve the nonlinear equations. The main idea of this algorithm is based on the exponential method. The proposed algorithm doesn’t have any derivative even though exponential method is involved, and moreover it converges fast. Numerical examples are presented to illustrate and validation of the proposed methods. Implementation of the proposed algorithm in Excel and Maple is discussed with sample computations.

## Limitations

In this paper, we focused on MS Excel and Maple implementation. However, the proposed algorithms can be implemented in many mathematical software tools such as Mathematica, SCILab, Matlab, etc.

## Data Availability

The datasets generated and analyzed during the current study are presented in this manuscript.

## Abbreviation

MS Excel: Microsoft Excel

## References

[1] Kincaid DE, Cheney EW (1990). Numerical analysis.

[2] Saqib M, Iqbal M, Ali S, Ismaeel T (2015). New fourth and fifth-order iterative methods for solving nonlinear equations. Appl Math.

[3] Waals VD, Diderik J. Over de continuiteit van den gas-en vloeistoftoestand (on the continuity of the gas and liquid state, Ph.D. dissertation, Leiden Univ., Leiden, The Netherlands; 1873.

[4] Hussain S, Srivastav VK, Thota S (2015). Assessment of interpolation methods for solving the real life problem. Int J Math Sci Appl.

[5] Thota S, Gemechu T, Shanmugasundaram P (2021). New algorithms for computing non-linear equations using exponential series. Palestine J Math.

[6] Thota S, Srivastav VK (2018). Quadratically convergent algorithm for computing real root of non-linear transcendental equations. BMC Res Notes.

[7] Thota S, Srivastav VK (2014). Interpolation based hybrid algorithm for computing real root of non-linear transcendental functions. Int J Math Comput Res.

[8] Thota S (2019). A new root-finding algorithm using exponential series. Ural Math J.

[9] Gemechu T, Thota S (2020). On new root finding algorithms for solving nonlinear transcendental equations. Int J Chem Math Phys.

[10] Parveen T, Singh S, Thota S, Srivastav VK. A new hydride root-finding algorithm for transcendental equations using bisection, regula-Falsi and Newton-Raphson methods. In: National conference on sustainable & recent innovation in science and engineering (SUNRISE-19); 2019. ISBN No. 978-93-5391-715-9.

[11] Srivastav VK, Thota S, Kumar M (2019). A new trigonometrical algorithm for computing real root of non-linear transcendental equations. Int J Appl Comput Math.

[12] Thota S. A new hybrid halley-false position type root finding algorithm to solve transcendental equations. In: Istanbul international modern scientific research congress–III, 06–08 May 2022, Istanbul Gedik University, Istanbul, Turkey.

[13] Thota S. A new three-step root-finding algorithm and its implementation in excel. In: International conference on evolution in pure and applied mathematics (ICEPAM-2022), 16–18 November; 2022, Department of Mathematics, Akal University, Talwandi Sabo, Punjab, India.

[14] Thota S, Kalyani P, Neeraj G, Balarama Krishna C. A new hybrid root-finding algorithm for solving transcendental equations using exponential and regula-falsi method. In: Virtual international conference on computational intelligence, simulation, financial engineering and mathematical modelling for industry and commerce, 30–31 August 2022, Great Zimbabwe University, Masvingo Zimbabwe.

[15] Thota S (2022). Solution of generalized Abel’s integral equations by homotopy perturbation method with adaptation in laplace transformation. Sohag J Math.

[16] Thota S, Shanmugasundaram P (2022). On new sixth and seventh order iterative methods for solving non-linear equations using homotopy perturbation technique. BMC Res Notes.

[17] Thota S, Maghrabi L, Shanmugasundaram P, Kanan M, Al-Sherideh AS (2023). A new hybrid root-finding algorithm to solve transcendental equations using arcsine function. Inf Sci Lett.

[18] Thota S, Ayoade AA (2022). On Solving Transcendental Equations Using Various Root Finding Algorithms with Microsoft Excel.

[19] Thota S. Microsoft excel implementation of numerical algorithms for nonlinear algebraic or transcendental equations. In: 5th international conference on statistics, mathematical modelling and analysis (SMMA 2022), May 27–29; 2022 in Xi’an, China.

[20] Thota S, Gemechu T, Ayoade AA (2023). On new hybrid root-finding algorithms for solving transcendental equations using exponential and Halley’s methods. Ural Math J.

[21] Noor MA, Khan WA, Hussain A (2007). A new modified Halley method without second derivatives for nonlinear equation. Appl Math Comput.

[22] Grau-Sanchez M, Gutierrez JM (2010). Some variants of the Chebyshev-Halley family of methods with fifth order of convergence. Int J Comput Math.

[23] Zhanlav T, Chuluunbaatar O, Ankhbayar G (2010). On newton-type methods with fourth and fifth-order convergence. Discrete Contin Models Appl Comput Sci.

[24] Nazeer W, Tanveer M, Kang SM, Naseem A (2016). A new Householder’s method free from second derivatives for solving nonlinear equations and polynomiography. J Nonlinear Sci Appl.

[25] Naseem A, Rehman MA, Abdeljawad T (2021). Real-world applications of a newly designed root-finding algorithm and its polynomiography. IEEE Access.

[26] Thota S (2019). A symbolic algorithm for polynomial interpolation with Stieltjes conditions in maple. Proc Inst Appl Math.

[27] Thota S. On a third order iterative algorithm for solving non-linear equations with maple implementation. In: National E-conference on interdisciplinary research in science and technology, May 30–31; 2020, Amiruddaula Islmia Degree College, Locknow, India.

[28] Rajawat RS, Singh KK, Mishra VN (2023). Approximation by modified Bernstein polynomials based on real parameters. Math Found Comput.

[29] Pandey S, Rajawat RS, Mishra VN (2023). Approximation properties of modified Jain-Gamma operators preserving linear function. Palestine J Math.

[30] Raiz M, Kumar A, Mishra VN, Rao N (2022). Dunkl analogue of Szasz Schurer beta operators and their approximation behavior. Math Found Comput.

[31] Ayoade AA, Thota S (2023). Functional education as a nexus between agricultural and industrial revolution: an epidemiological modelling approach. Uniciencia.

[32] Dubey R, Mishra LN, Ali R (2019). Special class of second-order nondifferentiable symmetric duality problem with (G, α_f_ )-pseudobonvexity assumptions. Mathematics.

[33] Auwalu A, Hincal E, Mishra LN (2019). On some fixed point theorems for expansive mappings in dislocated cone metric spaces with banach algebras. J Math Appl.

[34] Mishra LN, Raiz M, Rathour L, Mishra VN (2022). Tauberian theorems for weighted means of double sequences in intuitionistic fuzzy normed spaces. Yugoslav J Oper Res.

[35] Sharma MK, Dhiman N, Kumar S, Rathour L, Mishra VN (2023). Neutrosophic Monte Carlo simulation approach for decision making in medical diagnostic process under uncertain environment. Int J Neutrosophic Sci.

[36] Babolian E, Biazar J (2002). On the order of convergence of adomain method. Appl Math Comput.

[37] Traub JF (1982). Iterative methods for the solution of equations.

